# Plasma from patients with anti-glomerular basement membrane disease could recognize microbial peptides

**DOI:** 10.1371/journal.pone.0174553

**Published:** 2017-04-14

**Authors:** Jian-nan Li, Xiaoyu Jia, Yongqiang Wang, Can Xie, Taijiao Jiang, Zhao Cui, Ming-hui Zhao

**Affiliations:** 1 Renal Division, Department of Medicine, Peking University First Hospital, Beijing, China; 2 Institute of Nephrology, Peking University, Beijing, China; 3 Key Laboratory of Renal Disease, Ministry of Health of China, Beijing, China; 4 Key Laboratory of CKD Prevention and Treatment, Ministry of Education of China, Beijing, China; 5 Key Laboratory of Protein and Peptide Pharmaceuticals, Institute of Biophysics, Chinese Academy of Sciences, Beijing, China; 6 State Key Laboratory of Membrane Biology, Laboratory of Molecular Biophysics, School of Life Sciences, Peking University, Beijing, China; 7 Center for Systems Medicine, Institute of Basic Medical Sciences, Chinese Academy of Medical Sciences & Peking Union Medical College, Beijing, China; 8 Suzhou Institute of Systems Medicine, Suzhou, Jiangsu, China; 9 Peking-Tsinghua Center for Life Sciences, Beijing, China; Instituto Nacional de Ciencias Medicas y Nutricion Salvador Zubiran, MEXICO

## Abstract

Infection has long been suspected as a trigger of autoimmune diseases, and molecular mimicry mechanism was hypothesized in this study. Microbe originated peptides were searched from the Uniprot database based on a previous defined critical amino acid motif within α3_129−150_, isoleucine137, tryptophan140, glycine142, phenylalanine 143 and phenylalanine 145. 23826 microbial peptides were identified using our searching strategy, among which seven were related with human infections. Circulating IgG and IgM antibodies against the seven microbial peptides were detected using ELISA in 76 patients with anti-GBM disease. Four peptides were recognized by both IgG and IgM antibodies, and one peptide was recognized by IgG antibodies only. Peptides from Bacteroides, Saccharomyces cerevisiae, and Bifidobacterium thermophilum possessed the highest recognition frequency with the prevalence of 73.7%, 61.8% and 67.1% for IgG, 56.6%, 44.7% and 67.1% for IgM in anti-GBM patients. Patients with antibodies against these microbial peptides showed more severe kidney injury, including higher serum creatinine and higher percentage of crescent formation. In conclusion, antibodies against microbial peptides were identified in the circulation of anti-GBM patients, implying its etiological role in eliciting autoimmune response against α3(IV)NC1 through molecular mimicry.

## Introduction

Anti-glomerular basement membrane (GBM) disease, also called Goodpasture’s disease, is an autoimmune disorder characterized by the presence of anti-GBM autoantibodies, resulting in rapidly progressive glomerulonephritis and severe lung hemorrhage, with a high frequency of end stage renal disease (ESRD)[1–3]. The auto-antigen is non-collagenous domain 1 of α3 chain of type IV collagen [α3(IV)NC1][4, 5], with two major epitopes, E_A_ and E_B_[6]. Circulating antibodies and antigen-specific T cells have been proven to be pathogenic[7–10]. However, the mechanism on how autoantibodies and auto-reactive T cells generated is still unclear. In clinical practice, physicians found up to 50% of patients with anti-GBM disease suffered from prodromal infections before disease onset[11], which suggests that infections might act as one of the etiological events, or ‘second hits’ in human anti-GBM disease.

Molecular mimicry has been hypothesized in autoimmune diseases[12–23], such as anti-neutrophil cytoplasmic antibody associated vasculitis (AASV)[14, 19, 24–26], systemic lupus erythematosus (SLE)[12, 20] and multiple sclerosis (MS)[16]. However, the evidence of molecular mimicry in human anti-GBM disease is limited[27].

Arends J et al defined a T cell epitope (pCol(28–40), or α3_28−40_) on Goodpasture autoantigen in Wistar Kyoto (WKY) rats, and further identified its key amino acid residues[27, 28]. Then they immunized WKY rats with microbe originated peptides mimicking this T cell epitope, and these peptides successfully induced lung hemorrhage and glomerulonephritis in WKY rats, which provided the first experimental evidence of molecular mimicry in anti-GBM disease.

People with HLA-DRB1*1501 is reported to be susceptible to anti-GBM disease[29–34]. Using HLA-DRB1*15:01 transgenic mice, Ooi JD, et al defined an HLA-DRB1*1501-restricted T cell epitope, α3_136–146_[31]. Four amino acid residues, valine (V)_138_, tryptophan (W)_141_, glycine (G)_143_, and phenylalanine (F)_144_ were identified as the critical amino acid motif on this epitope. The corresponding amino acid residues on human α3(IV)NC1 were isoleucine (I)_137_, W_140_, G_142_, and F_143_. In our recent study, we identified P14 (α3_127–148_), as one of the major linear epitopes for B cells in patients with anti-GBM disease, which contains overlapped sequence with the above murine T cell epitope[11]. Three residues, G_142_, F_143_, and F_145_, (GFxF) on α3(IV)NC1 were identified as the critical amino acid motif of P14[35]. Taken together, we proposed that I_137_, W_140_, G_142_, F_143_, and F_145_, (IxxWxGFxF) was the most important critical amino acid motif on human α3(IV)NC1 for inducing cellular or humoral autoimmunity in anti-GBM patients.

In our present investigation, we identified seven microbe-derived peptides based on the motif (IxxWxGFxF) from Uniprot database, which could infect human beings. Both IgG and IgM antibodies against most of these peptides were detected in plasma from patients with anti-GBM disease. This result raised the possibility that epitope mimicry by microbial peptides might contribute to the onset of human anti-GBM disease.

## Results

### General data of patients

The demographic and clinical data of 76 patients with anti-GBM disease are shown in Table 1, 57.9% (44/76) of the patients had symptomatic infection before the onset of disease. 17 (22.4%) patients had coexistence of MPO-ANCA, whom are called double positive patients. 50 patients underwent sequence based genotype, among whom 39 (78%) patients carried HLA-DRB1*1501 allele.

**Table 1:** Clinical data of patients with anti-GBM disease.

Parameters	N = 76
Age (years)	40.6±18.2
Gender (male/female)	47/29
Prodromal infection, n (%)	44(57.9)
Hydrocarbon exposure, n (%)	10(13.2)
Smoking, n (%)	35(46.1)
Hemoptysis, n (%)	30(39.5)
Hemoglobin (g/L)	86.7±22.7
Oliguria/anuria, n (%)	30(39.5)
Urinary protein (g/24 h)	2.4(1.2, 5.2)
Nephrotic syndrome, n (%)	22(28.9)
Gross hematuria, n (%)	21(27.6)
Serum creatinine on diagnosis (μmol/L)	541.2(235, 885.4)
Anti-GBM antibody(IU)	175.0, 50.3–200
Positive ANCA, n (%)	17(22.4)
Crescents in glomeruli (%)	79.2±24.4
Renal survival at 1 yr, n (%)	13(19.4)
Patient survival at 1 yr, n (%)	58(86.6)

### Searching microbial peptides using the critical residues on α3(IV)NC1

We searched the Uniprot database (http://www.uniprot.org) for microbial peptide candidates that mimic the critical motif of isoleucine (I)_137_, tryptophan (W)_140_, glycine (G)_142_, phenylalanine (F)_143_, and phenylalanine (F)_145_ on α3(IV)NC1. Peptides were selected based on three criteria: 1) peptides with critical residues IxxWxGF; or 2) peptides with critical residues GFxF; and 3) peptides derived from microbes that could infect human beings. 23826 peptides were identified using our searching strategy, among which seven were related with human infections (Table 2), and were synthesized for further study (designated as P1-P7).

**Table 2:** Sequences of microbial peptides and their origins.

	Sequence	Protein (species)
P1	GATIKLWKGFSFRSTVGMR	TonB-linked outer membrane protein
		[Bacteroides sp. D22]
P2	DCIIINYWKGFIFSFHSYFFPF	Uncharacterized protein YEL014C
		[Saccharomyces cerevisiae (strain ATCC 204508/S288c)]
P3	NAAWKGFDFILFGTGSQ	TonB-dependent Receptor Plug
		domain-containing protein 894–905
		[Bacteroides sp. 4_1_36]
P4	RQALLWKGFSFSWYGTVFHDF	Binding-protein dependent transport system inner membrane protein
		[Bifidobacteriumthermophilum RBL67]
P5	QGFSFIMLFT	Bicyclomycin transporter TcaB
		[Staphylococcus warneri]
P6	LGFSFIMFVI	Cytochrome c oxidase subunit I
		[Stenotrophomonasmaltophilia JV3]
P7	SQMLWGFSFAMFT	Polysaccharide biosynthesis protein
		[Bacteroidetes oral taxon 274 str. F0058]

### Frequency of serum antibodies recognizing microbial peptides

**Fig 1:**
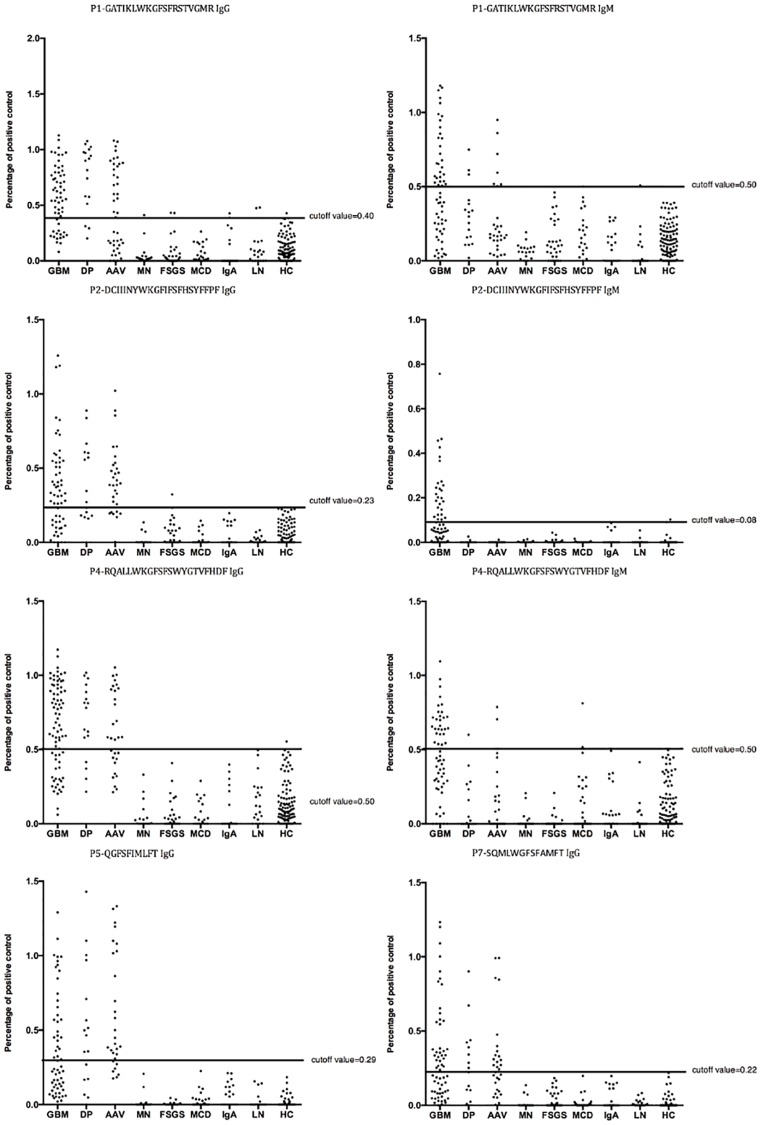
Levels of IgG and IgM antibodies against microbial peptides from patients with anti-GBM disease (GBM), Double Positive patients (DP), ANCA-Associated Vasculitis (AAV), Idiopathic Membranous Nephropathy (iMN), primary Focal Segmental Glomerulosclerosis (FSGS), Minimal Change Disease(MCD), IgA Nephropathy (IgAN), Lupus Nephritis (LN), and Healthy Controls (HC).

The frequencies and levels of antibodies against the seven microbial peptides are shown in Fig 1 and Table 3. 90.8% (69/76) of the anti-GBM patients had circulating IgG against at least one peptide, and 82.9% (63/76) had IgM. Among the seven peptides, plasma from anti-GBM patients was detected with IgG antibody reactions against 5/7 (71.4%) peptides, while IgM antibody reactions were detected against 4/7 (57.1%) peptides. P1, P2, P4, and P7 possessed both IgG and IgM reactions in patients, while P5 had only IgG reactions and P3 and P6 showed no reaction.

**Table 3:** The frequencies and levels of serum antibodies against microbial peptides.

Name	Sequence from N terminus	IgG antibodies, n, (%)	Mean levels of antibodies	IgM antibodies, n, (%)	Mean levels of antibodies
P1	GATIKLWKGFSFRSTVGMR	56 (73.7)	0.71±0.19	43 (56.6)	0.82±0.21
P2	DCIIINYWKGFIFSFHSYFFPF	47(61.8)	0.52±0.26	34 (44.7)	0.23±0.15
P3	NAAWKGFDFILFGTGSQ	0 (0)	-	0 (0)	-
P4	RQALLWKGFSFSWYGTVFHDF	51 (67.1)	0.84±0.17	51 (67.1)	0.70±0.13
P5	QGFSFIMLFT	41(53.9)	0.65±0.28	0 (0)	-
P6	LGFSFIMFVI	0 (0)	-	0 (0)	-
P7	SQMLWGFSFAMFT	41 (53.9)	0.51±0.28	5 (6.6)	0.17±0.06
P14	TDIPPCPHGWISLWKGFSFIMF	29 (38.2)	0.67±0.30	2 (2.6)	-

N = 76 patients

Circulating IgG and IgM antibodies against these peptides were also detected in patients with ANCA associated vasculitis (AASV) (67.4%(29/43)-IgG and 23.3%(10/43)-IgM, respectively) (Table 4). P1-IgG was detected in 72.9% (43/59) of patients with anti-GBM disease alone, 51.2% (22/43) of patients with AASV, and 82.4% (14/17) of double positive patients (*P* = 0.02). P2-IgG was detected in 57.6% (34/59) of patients with anti-GBM disease alone, 44.2% (19/43) of AASV patients, and 52.9% (9/17) of double positive patients (*P* = 0.41). P4-IgG was detected in 64.4% (38/59) of anti-GBM patients alone, 53.5% (23/43) of AASV patients, and 76.5% (13/17) of double positive patients (*P* = 0.23). P5-IgG was detected in 49.1% (29/59) of anti-GBM patients alone, 53.5% (23/43) of AASV patients, 70.6% (12/17) of double positive patients (*P* = 0.30). P7-IgG was detected in 52.5% (31/59) of anti-GBM patients alone, 37.2% (16/43) of AASV patients, and 58.8% (10/17) of double positive patients (*P* = 0.19).

**Table 4:** The comparison of anti-microbial peptide antibodies among patients with anti-GBM disease, ANCA associated vasculitis, and double positive patients.

	IgG			P value	IgM			P value
	Anti-GBM disease	ANCA associated vasculitis	Double positive		Anti-GBM disease	ANCA associated vasculitis	Double positive	
P1	72.9% (43/59)	51.2% (22/43)	82.4% (14/17)	0.02	64.4% (38/59)	9.3% (4/43)	47.1% (8/17)	<0.001
P2	57.6% (34/59)	44.2% (19/43)	52.9% (9/17)	0.41	57.6% (34/59)	14.0% (6/43)	0	<0.001
P3	0	0	0	-	0	0	0	-
P4	64.4% (38/59)	53.5% (23/43)	76.5% (13/17)	0.23	79.7% (47/59)	4.7% (2/43)	23.5% (4/17)	<0.001
P5	49.1% (29/59)	53.5% (23/43)	70.6% (12/17)	0.30	0	0	0	-
P6	0	0	0	-	0	0	0	-
P7	52.5% (31/59)	37.2% (16/43)	58.8% (10/17)	0.19	6.8% (4/59)	4.7% (2/43)	5.9% (1/17)	0.53

Circulating IgG and IgM antibodies against these peptides were also detected in patients with idiopathic membranous nephropathy (IgG: 1/18, 5.6%; IgM: 1/18, 5.6%), primary focal segmental glomerular sclerosis (IgG: 2/32, 6.3%; IgM: 7/32, 21.9%), minimal change disease (IgM: 1/21, 4.8%), IgA nephropathy (IgG: 2/20, 10%; IgM: 0/20, 5%), and lupus nephritis (IgG: 2/20, 10%; IgM: 1/20, 5%).

In order to confirm the specificity of circulating autoantibodies against microbial peptides in anti-GBM patients, antibodies against 20 linear peptides unrelated to microbes or α3(IV)NC1 were also detected. Only 2 of the 20 peptides were recognized by 3 of the 76 (3.9%) plasma samples.

Among the 39 patients with positive HLA-DRB1*1501, antibody reactions towards the seven microbial peptides were re-calculated and compared to those 11 patients without this susceptible HLA allele. No significant difference of recognition frequencies was identified between the two groups of patients (*P*>0.05).

### Clinical associations of antibodies recognizing microbial peptides

**Fig 2:**
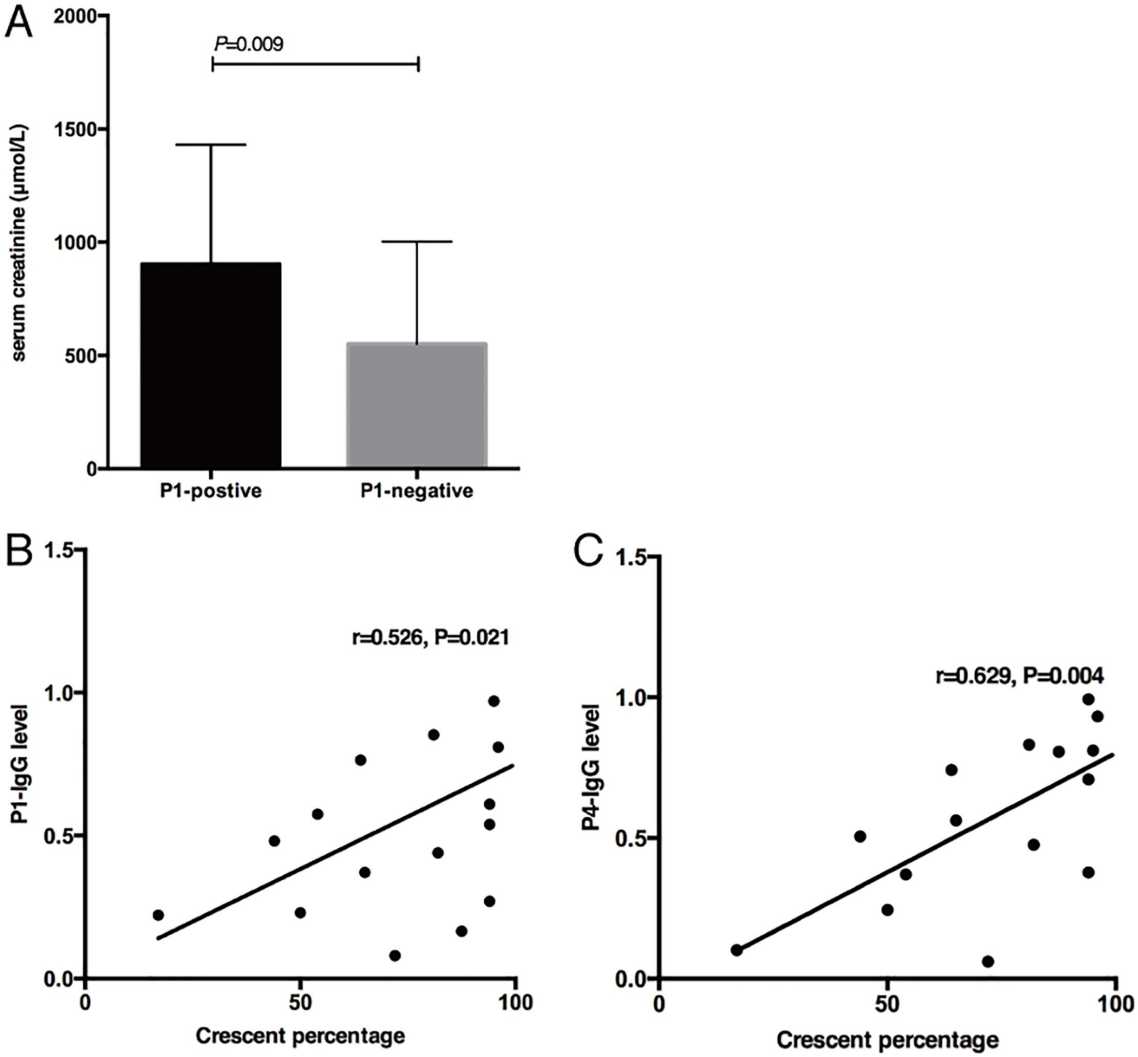
Clinical associations of antibodies against microbial peptides. A. Patients with anti-GBM disease with positive P1-IgG showed higher serum creatinine on diagnosis. B, C: Correlation between the level of P1-IgG or P4-IgG in anti-GBM patients with positive HLA-DRB1*1501 and the percentage of glomerular crescents.

In anti-GBM patients without MPO-ANCA, the patients having P1-IgG showed higher serum creatinine (800.0, 437.5–1500 vs. 312.8, 216.1–564.8μmol/L, *P* = 0.009) compared to those without P1-IgG (Fig 2A). The level of P1-IgG was positively correlated with serum creatinine (*r* = 0.292, *P* = 0.03). The level of P4-IgG and P5-IgG were positively correlated with the age of patients (*r* = 0.312, *P* = 0.02; *r* = 0.311, *P* = 0.02, respectively). The titer of autoantibody against P1 was positively correlated with that of α3(IV)NC1 (P = 0.041, r = 0.234). No significant association was found between the titer of autoantibodies against other peptides and α3 or P14.

In 39 anti-GBM patients carrying HLA-DRB1*1501 allele, the patients having P1-IgG had higher percentages of crescent formation in glomeruli (96, 81.5-100vs. 68.5, 41.8–89.1%, *P* = 0.03). Levels of P1-IgG and P4-IgG were positively correlated with the percentages of crescent formation, respectively (P1-IgG: *r* = 0.526, *P* = 0.02; P4-IgG: *r* = 0.629, *P* = 0.004, Fig 2B and 2C). Patients with P2-IgG had a higher prevalence of prodromal infections before the disease onset (79.2% vs.33.3%, *P* = 0.01).

Among the 43 anti-GBM patients with MPO-ANCA, the patients with positive P2-IgM had a higher level of serum C reaction protein (CRP) [151.0, 93.2–162.0 vs. 11.4, 4.2–52.0 mg/l, *P* = 0.002]. Patients with positive P1-IgG and P5-IgG showed a lower level of hemoglobin [P1-IgG: 7.1, 5.6–7.6 vs. 8.6, 7.1–9.4 g/dl, *P* = 0.02; P5-IgG: 7.1, 5.7–8.2 vs. 8.6, 7.5–9.6 g/dl, *P* = 0.02], respectively. The level of P5-IgG was positively correlated with 24-hour urinary protein excretion (*r* = 0.42, *P* = 0.02). The level of P4-IgG was positively correlated with erythrocyte sedimentation rate (ESR) (*r* = 0.35, *P* = 0.03).

Anti-GBM patients with positive P2-IgM and P4-IgM had a lower rate of positive ANCA (P2-IgM: 0 vs. 40%, *P*<0.001; P4-IgM: 7.8% vs. 52%, *P*<0.001), respectively. The level of P1-IgG in double positive patients was positively correlated with the percentage of crescents on renal biopsy (*r* = 0.936, *P* = 0.006).

### Cross-reaction between autoantibodies against α3(IV)NC1, P14 and microbial peptides

**Fig 3:**
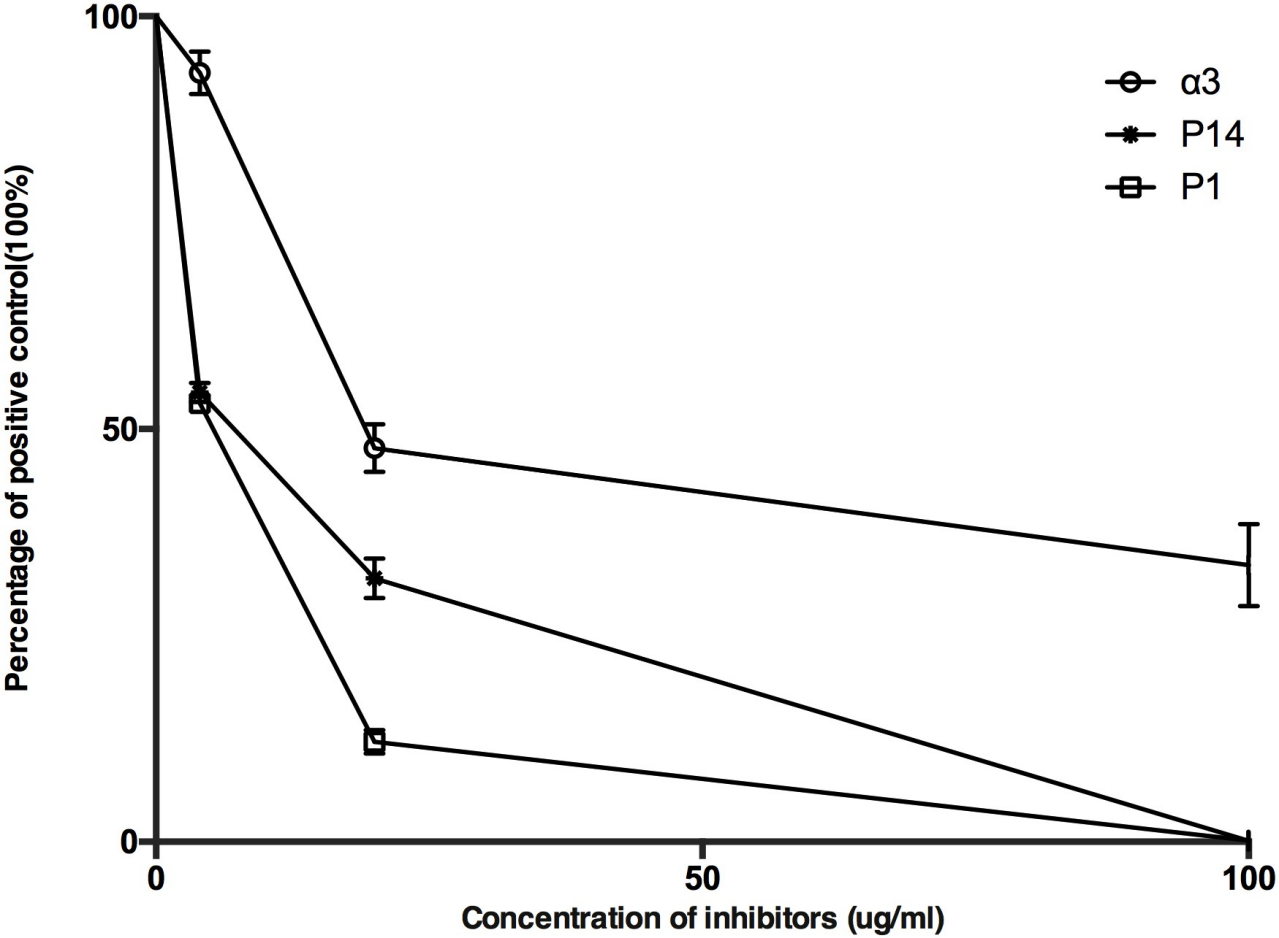
Competition assays of autoantibodies against microbial peptides. Antibody binding to P1 could be inhibited by P1, P14, and α3(IV)NC1. Binding to immobilized P1 was measured using ELISA, and relative binding is calculated as a percentage of antibody binding to immobilized P1-IgG in the absence of P1, P14 or α3(IV)NC1in solution.

An inhibition ELISA was performed to examine cross-reaction between autoantibodies against α3(IV)NC1 or P14 and the antibodies against microbial peptides. Binding of antibodies against the microbial peptides was strongly inhibited by themselves, as well as α3(IV)NC1 and P14, which indicated that the antibodies against microbial peptides cross reacted with P14 and α3(IV)NC1 (Fig 3).

## Discussion

The present study demonstrated that microbe-derived peptides with critical motif on α3(IV)NC1 could be recognized by the majority of anti-GBM patients. Their antibodies showed cross-reaction with those against P14 and α3(IV)NC1. These findings implicate that molecular mimicry might be a link between infection and autoimmunity in human anti-GBM disease.

In the current study, we found that four microbial peptides possessed both circulating IgM and IgG reactions and one possessed IgG reaction in anti-GBM plasma. All of them were derived from opportunistic bacteria. Although they may not induce symptomatic infections themselves, they might elicit immune responses when they gain access to the circulation[36], and the ensuing epitope spreading and somatic hypermutation[37–39] might cause autoimmunity towards P14 and α3(IV)NC1. This finding provided the first evidence for the activation of immune responses towards bacteria in the circulation of patients with anti-GBM disease, which is the first step of molecular mimicry-mediated autoimmune responses.

The titer of autoantibodies against these microbial peptides presented positive correlation with the severity of kidney damage. However, the level of autoantibodies against α3(IV)NC1 only correlated with that of P1 among the seven microbial peptides. In our previous study, we found that intermolecular epitope spreading happened in patients with severe renal damage, with higher titer of autoantibodies against different α chains[40]. Similarly, in this case, higher titer of autoantibodies against microbial peptides might indicated larger chance of epitope spreading to P14 and α3(IV)NC1. Whether these autoantibodies had direct pathogenic role in kidney damage needs further investigation.

78% of anti-GBM patients included in our study carried HLA-DRB1*1501 allele, which is consistent with previous reports[29, 30, 33]. Although no significant difference on the frequency of microbial peptide recognition was found between the patients with and without HLA-DRB1*1501 allele, closer associations between antibodies against bacteria-derived peptides and kidney injury was observed in patients having HLA-DRB1*1501. HLA-DRB1*1501 is the susceptible allele for anti-GBM disease, therefore, epitope spreading from microbial peptides to P14 and α3(IV)NC1 might be easier to happen in patients with HLA-DRB1*1501 and resulting in more severe renal damage. Using microbial peptides based on critical motifs of α3, this cross reactivity was detected in patients with anti-GBM disease. However, further attempts were needed on how the cross-reactivity happened on T cell and B cell levels[41].

It is interesting that we detected autoantibody towards the microbial peptides both in plasma of patients with ANCA-associated vasculitis and anti-GBM disease, but few were detected in patients with other autoimmune kidney diseases, such as lupus nephritis, IgA nephropathy, or idiopathic membranous nephropathy. It has been reported that almost 30% patients with of anti-GBM disease had coexisting MPO-ANCA[42–45]. Since no cross reaction was found between MPO and GP antigen[44], cross detection in linear epitopes of MPO and GP autoantigen were explored in our previous investigation, which found that over half of anti-GBM patients had autoantibodies against linear peptides derived from MPO[46]. In our present investigation, there is no significant difference in the titer of IgG autoantibody against P2, P4, P5 and P7 and IgM autoantibody against P7 between AASV and anti-GBM patients. This interesting result suggests that in the initiation of the two diseases, similar microbes generated immune response in the hosts, but different genetic predispositions, especially HLA alleles, lead to different antigen presentation and epitope spreading process subsequently[47, 48].

This observational study has several limitations. Firstly, although antibodies towards microbial peptides were found in patients, immunization of these peptides failed to induce crescentic glomerulonephritis in WKY rats. Therefore, further investigations in humanized mice models might be needed to further elucidate the pathogenicity of these microbial peptides. Second, microbe-derived peptide searching was only based on the critical amino acids on α3_136–146_, which may result in missing of other potential microbes mimicking other parts of the whole α3(IV)NC1.

In conclusion, antibodies towards microbe-derived peptides were detected in the circulation of patients with anti-GBM disease. These microbes might etiologically participate in the pathogenesis of human anti-GBM disease through molecular mimicry.

## Materials and methods

### Patients and plasma

76 patients with anti-GBM disease were included in Peking University First Hospital from 1996 to 2012. Clinical data were collected at the time of diagnosis as well as during follow-up. All the patients had positive circulating anti-GBM antibodies, as detected by ELISA using purified bovine α(IV)NC1 (Euroimmun, Lubeck, Germany) and recombinant human α3(IV)NC1 as solid-phase antigens. 17 out of the 76 patients were also positive for MPO-ANCA, detected by both indirect immunofluorescence assay and antigen-specific ELISA using purified MPO as solid phase ligand (Euroimmun, Lubeck, Germany). Plasma from patients with anti-GBM disease was collected before initiation of immunosuppressive therapy. Among the 76 patients, 50 patients had DNA samples and underwent HLA genotyping. Plasma from 18 patients with membranous nephropathy (MN), 32 patients with primary focal segmental glomerulosclerosis (FSGS), 21 patients with minimal change disease (MCD), 20 patients with IgA nephropathy, 20 patients with lupus nephritis were collected as disease controls.

Plasma from 91 healthy blood donors was collected as normal controls. All the plasma was stored at -20°C until use. The research was in compliance of the Declaration of Helsinki and approved by the ethics committee of Peking University First Hospital. Written informed consent was obtained from each participant. Authors do not have access to information that could identify individual participants after data collection.

### Searching microbial peptides using the critical residue motif on α3(IV)NC1

A T cell epitope, α3_136−146_, has been defined using HLA-DRB1*1501 transgenic mice, with critical amino acids as valine (V)_138_, tryptophan (W)_141_, glycine (G)_143_, and phenylalanine (F)_144_(27). The corresponding amino acid residues on human α3(IV)NC1 were isoleucine (I)_137_, tryptophan (W)_140_, glycine (G)_142_, and phenylalanine (F)_143_. We identified P14 (α3_127–148_) as one of the major linear epitopes for B cells in patients with anti-GBM disease(10) with three critical amino acids, glycine (G)_142_, phenylalanine (F)_143_, and phenylalanine (F)_145_(33). Taken together, the above five residues, isoleucine (I)_137_, tryptophan (W)_140_, glycine (G)_142_, phenylalanine (F)_143_(IxxWxGFxF), were used as the searching sequence for microbial peptides.

We searched the Uniprot database (http://www.uniprot.org) for microbial peptide candidates based on the critical motif of IxxWxGFxF. Peptides were selected based on three criteria: 1) peptides with critical residues IxxWxGF; or 2) peptides with critical residues GFxF; and 3) peptides derived from microbes were related to infections in human beings.

### Preparation of linear peptides

Peptides were synthesized on an automatic peptide synthesizer using Fmoc (9-fluorenyl-methyloxycarbonyl) chemistry (Beijing Scilight Biotechnology Ltd Co, Beijing, China), and purified by a reverse-phase CIS column on a preparative HPLC. Purified peptides were analyzed by HPLC for purity and mass spectrometry for correct sequence. Peptides with purity > 98% were used for further tests.

### Detection of circulating antibodies against peptides by ELISA

Highly purified synthetic peptides were diluted at 10μg/ml with coating buffer (0.05M bicarbonate buffer, pH 9.6) and plated on half of the wells of a polystyrene microtitre plate (Nunc Immunoplate; Nunc, Roskilde, Denmark), at 4°C overnight. The other half was coated with coating buffer alone as antigen-free wells to exclude non-specific binding. The plate was washed three times with PBS containing 0.1% Tween-20 (PBST) between steps. 2% bovine serum albumin (BSA) diluted with PBS was used to block non-specific binding sites. The plasma was diluted to 1:100 in PBST containing 1% BSA and were added in duplication. Every plate contained positive, negative and blank controls. The plate was incubated at 37°C for 60min. The binding was detected with alkaline phosphatase-conjugated goat anti-human IgG or IgM (Fc specific; Sigma, St Louis, MO, USA) at a dilution of 1:5000. P-nitrophenyl phosphate (1mg/ml; Sigma) was used in substrate buffer [1M diethanolamine and 0.5mM MgCl_2_ (pH 9.8)]. Color development was measured spectrophotometrically at 405nm (Bio-Rad, Tokyo, Japan) 30min later. Plasma from 91 healthy blood donors were used to build up the cutoff value using mean+2SD. The positive control was chosen as a sample with an OD value of 1.5. Each plate contained this positive control. Samples were re-examined when SEMs >10% were found.

### Cross-reaction between autoantibodies against α3(IV)NC1, P14 and microbe-derived peptides

Cross-reactions between autoantibodies against α3(IV)NC1 or P14 and microbial peptides were investigated using an inhibition ELISA. Briefly, polystyrene microtiter plates were coated with soluble microbial linear peptides (P1-P7) at a concentration of 10 μg/ml. Diluted plasma was pre-incubated with soluble linear peptides (P1-P7), recombinant human α3(IV)NC1 or P14 at a concentration from 0.5 to 50 μmol/l at 37°C for 60 min. The mixtures were then transferred to the peptide-coated microtiter plates, and the bound autoantibodies were detected with alkaline phosphatase-conjugated secondary autoantibodies, as described above.

### HLA allele genotyping

Peripheral blood samples (10ml) were collected from patients and controls using anticoagulant EDTA. Genomic DNA was obtained from peripheral blood leukocytes using the Puregene Blood Core Kit C (Qiagen Science, Germany). HLA-DRB1, -DQB1, and -DPB1 sequencing was performed on an Applied Biosystems 3130xl platform using SeCore^®^ Sequencing Kits (Invitrogen, Leek, Netherlands). HLA alleles typing reports were issued using uType SBT HLA software. HLA-DRB1, and -DQB1, and -DPB1 typing was performed via bidirectional sequencing of exon 2, exons 2 and 3, and exons 2, 3, and 4, respectively; HLA-DQA1 alleles were typed using electrophoresis.

### Statistical analysis

Differences in quantitative parameters were assessed using Student’s *t* test or nonparametric test. Differences in qualitative data were compared using chi-squared test or Fisher’s exact test. Pearson and Spearman rank correlations were performed to analyze the relationship between antibody levels and clinical data as appropriated. Peptides with recognition <5% were excluded from further statistical analysis. P value <0.05 was considered significant. Analysis was performed with SPSS statistical software package (version 10.0, Chicago, IL, US).
